# Electrically Charged Disinfectant Containing Calcium Hydrogen Carbonate Mesoscopic Crystals as a Potential Measure to Control *Xanthomonas campestris* pv. *campestris* on Cabbage Seeds

**DOI:** 10.3390/microorganisms8101606

**Published:** 2020-10-19

**Authors:** Akikazu Sakudo, Makoto Haritani, Koichi Furusaki, Rumiko Onishi, Takashi Onodera

**Affiliations:** 1 School of Veterinary Medicine, Okayama University of Science, Imabari, Ehime 794-8555, Japan; 2 Laboratory of Biometabolic Chemistry, School of Health Sciences, University of the Ryukyus, Nishihara, Okinawa 903-0215, Japan; 3 Laboratory of Environmental Science for Sustainable Development, Department of Global Agricultural Science, The University of Tokyo, Bunkyo-ku, Tokyo 113-8657, Japan; aharitani@g.ecc.u-tokyo.ac.jp (M.H.); atonode@g.ecc.u-tokyo.ac.jp (T.O.); 4 Mineral Activation Technical Research Center, Omuta, Fukuoka 836-0041, Japan; riken@jade.plala.or.jp; 5 Santa Mineral Co., Ltd., Minato-ku, Tokyo 105-0013, Japan; rumiko@santa-mineral.co.jp; 6 Research Center for Food Safety, The University of Tokyo, Bunkyo-ku, Tokyo 113-8657, Japan

**Keywords:** bacterium, black rot, food safety, germination, plant stem growth, seeds, *Xanthomonas campestris* pv. *campestris*

## Abstract

*Xanthomonas campestris* pv. *campestris* (*Xcc*) is an important seed-borne bacterial pathogen that causes black rot in brassica. Current seed disinfection methods for *Xcc* have disadvantages; chemical treatment has associated environmental risks, hot water immersion reduces germination, and dry heat treatment is protracted. Here, we treated *Xcc*-contaminated seeds with CAC-717, a recently developed disinfectant produced by applying an electric field and water flow to distilled water containing calcium hydrogen carbonate to produce mesoscopic crystals. The decimal reduction time (*D*-value) of *Xcc* suspension (8.22 log_10_ colony forming units (CFU)/mL) by CAC-717 treatment was 0.319 min. Treatment of *Xcc*-contaminated cabbage seeds at 25 °C for 30 min with CAC-717 significantly reduced bacterial cell numbers recovered from the seeds (0.36 log_10_ CFU/mL (SEM (standard error of the mean) = 0.23 log_10_ CFU/mL)) compared with distilled water treatment (3.52 log_10_ CFU/mL (SEM = 0.12 log_10_ CFU/mL)). Moreover, there was a lower incidence of black rot after treatment with CAC-717 (26.67% ± 3.33%) versus distilled water (56.67% ± 8.82%). For non-contaminated seeds, there was no significant difference in germination rate and plant stem length between distilled water and CAC-717 treatment after 5 days of cultivation. In conclusion, CAC-717 is a promising seed disinfectant without deleterious effects on germination or plant growth.

## 1. Introduction

*Xanthomonas campestris* pv. *campestris* (*Xcc*) is a pathogenic Gram-negative seed-borne bacterium [1] that causes black rot [2]. Black rot can lead to the extensive loss of cabbage and other cruciferous plants, which is a worldwide multibillion dollar industry [3]. Contaminated seeds and plant debris in soil as well as infected transplants are the main transmission routes of *Xcc* [1].

Black rot can be prevented and controlled by adopting various strategies such as using pathogen-free seeds, employing good sanitary practices, managing insects and weeds, planting varieties of crops with partial resistance to black rot, and using chemical control [4,5]. However, these measures have collectively failed to manage the disease [1,5,6].

Seed treatments for *Xcc* disinfection include chemicals such as calcium hypochlorite, sodium hypochlorite, hydrogen peroxide, and hot acidified cupric acetate or zinc sulphate, as well as physical treatments such as hot water immersion and dry heat, although none of these measures are totally effective [1,6,7,8]. In addition, these methods have disadvantages, such as decreasing the germination rate and increasing residual agricultural chemicals, and they involve a prolonged treatment time, among other disadvantages [6]. In particular, although 20,000 ppm calcium hypochlorite is recommended by the US Food and Drug Administration (FDA), it poses environmental and public safety risks [4,7]. Consequently, more sustainable and eco-friendly seed disinfection processes are required without sacrificing effectiveness.

Recently, we developed a new electrically charged disinfectant, termed CAC-717, by applying an electric field and water flow to distilled water containing calcium hydrogen carbonate [9]. CAC-717 contains particles (50–500 nm) with a mesoscopic structure of calcium hydrogen carbonate derived from calcium and carbon in plant and soil [9,10].

CAC-717 has been shown to have a bactericidal effect against *Escherichia coli* [10] and *Salmonella enterica* [10], as well as a virucidal effect on influenza virus [9], feline calicivirus (FCV) [10], and human/murine norovirus [11]. In addition, CAC-717 has no harmful effects on humans or animals because its pH decreases to 8.8 ± 1.2 immediately after contact with biological samples [9]. Thus, we believe that an alkaline pH is not a major factor associated with the inactivation effect of CAC-717. We have further extended these studies to determine whether CAC-717 can be applied as a safe and efficient seed disinfection technology.

In this study, we treated *Xcc*-contaminated seeds with CAC-717 under in vitro conditions and then evaluated disinfection efficiency and disease incidence. Based on our results, we discuss the effectiveness of CAC-717 for seed disinfection.

## 2. Materials and Methods

### 2.1. Preparation of Bacterial Cultures

Yeast extract–dextrose–CaCO_3_ (YDC) plate medium (about pH 7.0) comprising 10 g/L Bacto yeast extract (Becton, Dickinson and Company, Franklin Lakes, NJ, USA), 20 g/L calcium carbonate (Nacalai Tesque, Inc. Kyoto, Japan), 20 g/L *D*-glucose carbonate (Nacalai Tesque, Inc.), and 15 g/L Bacto Agar (Becton, Dickinson and Company) was used for the cultivation of *Xcc* (NGM120310-14 strain) [12]. After proliferation on YDC plate medium at 25 °C for 2 days, colonies of *Xcc* were collected and suspended in 1 mL of distilled water for use as a bacterial suspension.

### 2.2. Synthesis of the Electrically Charged Disinfectant (CAC-717)

**Figure 1:**
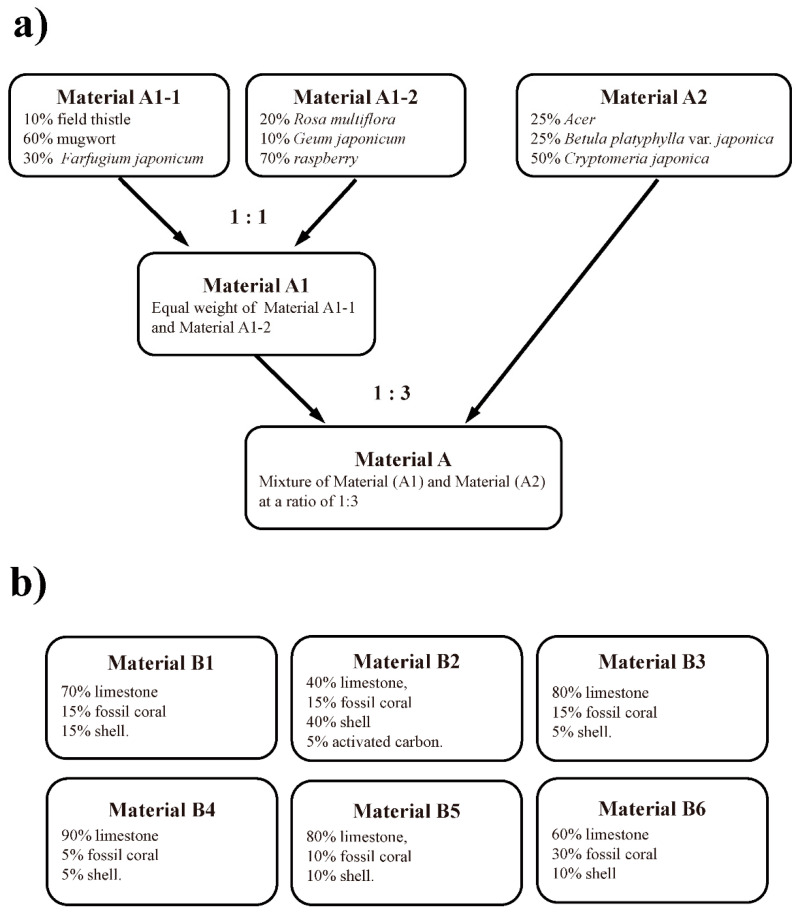
Preparation of Material (A) and Material (B1–B6). (**a**) To produce Material (A), Material (A1) was mixed with an equal weight of Material (A1-1) and Material (A1-2). Material (A1-1) was a dried and pulverized mixture of 10% (*w*/*w*) field thistle (leaf part, stem part, and flower part), 60% (*w*/*w*) mugwort (leaf part and stem part), and 30% (*w*/*w*) *Farfugium japonicum* (leaf part and stem part). Material (A1-2) was a dried and pulverized mixture of 20% (*w*/*w*) *Rosa multiflora* (leaf part, flower part), 10% (*w*/*w*) *Geum japonicum* (leaf part and stem part), and 70% (*w*/*w*) raspberry (leaf part, stem part, and flower part). Material (A2) was a dried mixture of 25% (*w*/*w*) *Acer* (leaf part and stem part), 25% (*w*/*w*) *Betula platyphylla* var. *japonica* (leaf part, stem part, and bark part), and 50% (*w*/*w*) *Cryptomeria japonica* (leaf part, stem part, and bark part). Then, Material (A1) and Material (A2) was mixed at a ratio of 1:3 to obtain Material (A). (**b**) To produce Material (B), various combinations of limestone, fossil coral, shell, and activated carbon were used to obtain Material (B1), Material (B2), Material (B3), Material (B4), Material (B5), and Material (B6). Material (B1) comprised 70% (*w*/*w*) limestone, 15% (*w*/*w*) fossil coral, and 15% (*w*/*w*) shell. Material (B2) comprised 40% (*w*/*w*) limestone, 15% (*w*/*w*) fossil coral, 40% (*w*/*w*) shell, and 5% (*w*/*w*) activated carbon. Material (B3) comprised 80% (*w*/*w*) limestone, 15% (*w*/*w*) fossil coral, and 5% (*w*/*w*) shell. Material (B4) comprised 90% (*w*/*w*) limestone, 5% fossil coral, and 5% shell. Material (B5) comprised 80% (*w*/*w*) limestone, 10% (*w*/*w*) fossil coral, and 10% (*w*/*w*) shell. Material (B6) comprised 60% (*w*/*w*) limestone, 30% (*w*/*w*) fossil coral, and 10% (*w*/*w*) shell.

CAC-717 (Food and Drug Administration/USA Regulation No. 880.6890 Class 1 disinfectant, approximate pH 12.4) [9,13], which contains 6.9 mM calcium hydrogen carbonate particles with a mesoscopic structure, was produced by mixing Solution (A) and Solution (B) at a 1:10 ratio, in accordance with Japan patent No. 5778328. Solution (A) and Solution (B) were prepared from Material (A) and Material (B1–B6) (Figure 1), as described below.

Briefly, to produce Solution (A), Material (A) was added to distilled water at 12.5% (*w*/*v*) in an apparatus described in Japan patent No. 5778328. Then, direct current (DC 8300 V, 100 mA) was applied to the conductive lines of the apparatus using a Teflon insulation-coated electrostatic field electrode (N-800N, Mineral Activation Technical Research Center, Kumamoto, Japan) together with water flow generated around the conductive wires in the same direction as the direct current as well as ultrasonic vibration (oscillation frequency, 50 kHz; amplitude, 1.5/1000 mm). Thereafter, the resultant solution was subjected to exposure to far-infrared radiation at a wavelength of 6–14 µm to obtain Solution (A). To produce Solution (B), distilled water was passed through six vessels containing Material (B1), Material (B2), Material (B3), Material (B4), Material (B5), and Material (B6), respectively, as described in Japan patent No. 5778328.

### 2.3. Treatment of Xcc with CAC-717, Distilled Water, or Hot Water, and the Evaluation of Bacterial Cell Number

An aliquot (20 µL) of *Xcc* bacterial suspension was mixed with 20 µL of CAC-717 and then incubated at 25 °C for 0, 0.5, 1, 2, or 5 min. As control, an aliquot (20 µL) of *Xcc* bacterial suspension was mixed with 20 µL of distilled water and then incubated at 25 °C or 50 °C for 0, 0.5, 1, 2, or 5 min, because hot water treatment (50 °C) of cabbage seeds is considered the standard method for disease management [6,14,15,16]. After treatment, the cell suspension was immediately diluted with 1 mL of distilled water and spread on a YDC plate. The number of CFU per mL was measured by counting colonies of *Xcc* after incubation for 3 days at 25 °C.

### 2.4. Determination of the Decimal Reduction Time (D-Value)

The *D*-value was defined as the time required to reduce the original number of viable cells by 90% [17] and was calculated by using the following equation: *D* = 1/(∆logN/∆t), where ∆t is the time for a one log_10_ reduction in viable *Xcc* cells, and ∆logN is the logarithmic value of the change in the CFU/mL of *Xcc* after CAC-717 treatment.

### 2.5. Preparation of Xcc-Contaminated Seeds and Non-Contaminated Seeds

*Xcc* cultured on a YDC plate at 25 °C for 2 days was suspended in 20 mL of sterilized distilled water containing 0.02% Tween 20. Cabbage seeds (Cabbage No. 1, Wakaba, Italian) were immersed in 70% ethanol for 2 min, rinsed with sterilized distilled water, and air-dried on sterilized filter paper. Thereafter, each of the 135 seeds were soaked in either 20 mL of *Xcc* suspension (10 log_10_ CFU/mL) or sterilized distilled water for 1 h and air-dried on filter paper to obtain *Xcc*-contaminated seeds or non-contaminated seeds, respectively.

### 2.6. CAC-717 Treatment of Seeds

Ten seeds were each incubated in 1 mL of CAC-717 at 25 °C for 30 min. The treatment time of 30 min was chosen because this corresponds to the conventional disinfection method for *Xcc*-contaminated cabbage seeds [6,15,16]. A control treatment using distilled water instead of CAC-717 was also performed under identical conditions. These analyses were performed in triplicate, and the experiment was subsequently repeated (i.e., in all, 60 seeds were analyzed for each group).

### 2.7. Collection of Bacteria from Xcc-Contaminated Seeds

Each of the 5 *Xcc*-contaminated seeds that had been subjected to either distilled water treatment or CAC-717 treatment were suspended in sterilized distilled water (500 µL) and then vortexed for 30 s. Next, the seeds were allowed to settle by gravity after incubation for 1 min. The resultant supernatants were plated on YDC medium, and the number of viable bacterial cells were counted after incubation for 3 days. These analyses were performed in triplicate, and the experiment was subsequently repeated (i.e., in all, 30 seeds were analyzed for each group).

### 2.8. Calculation of Disease Incidence

To analyze the incidence of disease, two sterilized filter papers were placed in a sterile Petri dish (diameter 100 mm), and then 5 mL of sterilized distilled water was added dropwise. Ten seeds were arranged on the paper at equal intervals and cultured at 25 °C for 5 days. This cultivation time was chosen because the first symptoms of *Xcc* contamination appear on the cotyledons after this time point. The incidence of disease among the 10 seedlings was calculated by assessing the wilting of cotyledons and yellowing of leaves as an index of disease onset.

### 2.9. Calculation of Germination Rate and Plant Stem Length Measurement

Seeds treated with CAC-717 or distilled water as described above were cultured at 25 °C for 5 days. The germinated and growing cabbage was photographed. Germination rate was then determined. The length of each plant stem was measured using ImageJ software v. 1.52a (National Institute of Health, Bethesda, MD, USA).

### 2.10. Statistical Analysis

Experiments were performed in triplicate. Differences between control treatment (0 min) and CAC-717 treatment at various time points were assessed by non-repeated analysis of variance (ANOVA) followed by Bonferroni’s multiple comparison test. In the experiments on cabbage seeds, differences between control (distilled water) treatment and CAC-717 treatment for 30 min were assessed by Mann–Whitney U-test. All statistical analyses were carried out using GraphPad Prism 7 software (GraphPad Prism Software Inc., La Jolla, CA, USA).

## 3. Results

**Figure 2:**
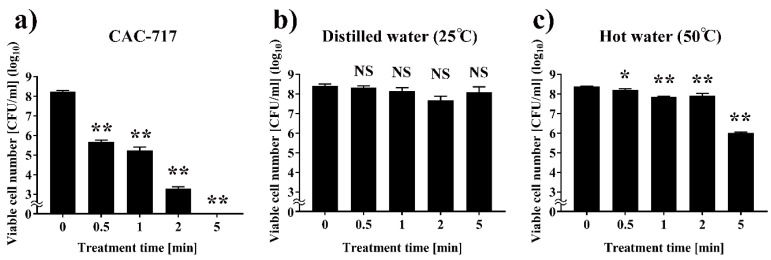
Reduction of viable cell number of *Xanthomonas campestris* pv. *campestris* (*Xcc*) after CAC-717 treatment. *Xcc* suspension (8.22 log_10_ colony forming units (CFU)/mL) was mixed with an equal quantity of CAC-717 (**a**) and incubated at 25 °C for the indicated times (0, 0.5, 1, 2, or 5 min). As a control, *Xcc* suspension mixed with an equal quantity of distilled water was subjected to incubation at 25 °C (**b**) or 50 °C (**c**) for the indicated times (0, 0.5, 1, 2, or 5 min). After treatment, the samples were plated on yeast extract–dextrose–CaCO_3_ (YDC) medium at 25 °C for 3 days, and the bacterial cell number was determined as colony forming units (CFU)/mL. Data shown as mean ± SEM (standard error of the mean) of triplicates and are representative of two independent experiments; * and ** indicate significant differences (*p* < 0.05 and *p* < 0.01, respectively) versus the control (0 min) by non-repeated measured analysis of variance (ANOVA) followed by Bonferroni correction. NS: no significant difference versus control (0 min).

First, the number of viable cells of *Xcc* after treatment with CAC-717 was investigated (Figure 2). The results showed that the initial viable cell number of *Xcc* (0 min) was 8.22 log_10_ CFU/mL (SEM (standard error of the mean) = 0.08 log_10_ CFU/mL) colony forming units (CFU)/mL. Treatment with CAC-717 caused a significant decrease in cell number to 5.63 log_10_ CFU/mL (SEM = 0.13 log_10_ CFU/mL) after 0.5 min, 5.09 log_10_ CFU/mL (SEM = 0.31 log_10_ CFU/mL) after 1 min, 3.27 log_10_ CFU/mL (SEM = 0.09 log_10_ CFU/mL) after 2 min, and below the detection limit after 5 min (Figure 2a). From these data of the viable cell number, we calculated the treatment time required for achieving a 90% reduction of viable cell number (*D*-value) by CAC-717 treatment to be 0.319 min. Thus, as a control, *Xcc* was subjected to treatment with distilled water (25 °C) or hot water (50 °C) in place of CAC-717. The results showed no significant reduction of viable cell number by distilled water treatment within 5 min compared to 0 min (Figure 2b). Hot water (50 °C) treatment showed a slight but significant decrease in viable cell number of *Xcc* (Figure 2c). However, the *D*-value of hot water (50 °C) treatment was 2.137 min, which was much greater than the value determined for CAC-717 treatment.

**Figure 3:**
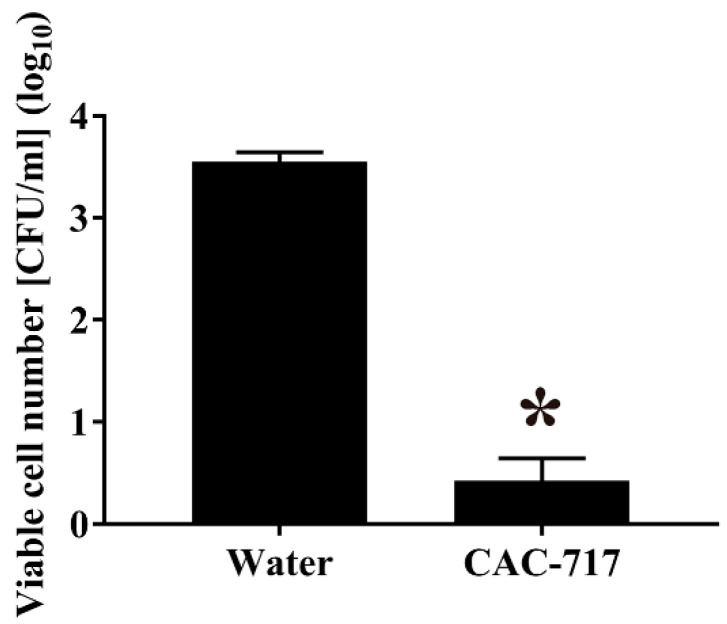
Reduction of viable cell number of *Xcc* in *Xcc*-contaminated seeds after CAC-717 treatment. Cabbage seeds contaminated with *Xcc* were treated with distilled water or CAC-717 at 25 °C for 30 min, and *Xcc* was recovered from the seeds as described in Methods. The samples were then plated and incubated on YDC medium at 25 °C, and the viable cell number (CFU/mL) was counted after 3 days. Data shown as mean ± SEM of triplicates and are representative of two independent experiments; * indicates a significant difference (*p* < 0.05) between the two groups by Mann–Whitney U-test.

**Figure 4:**
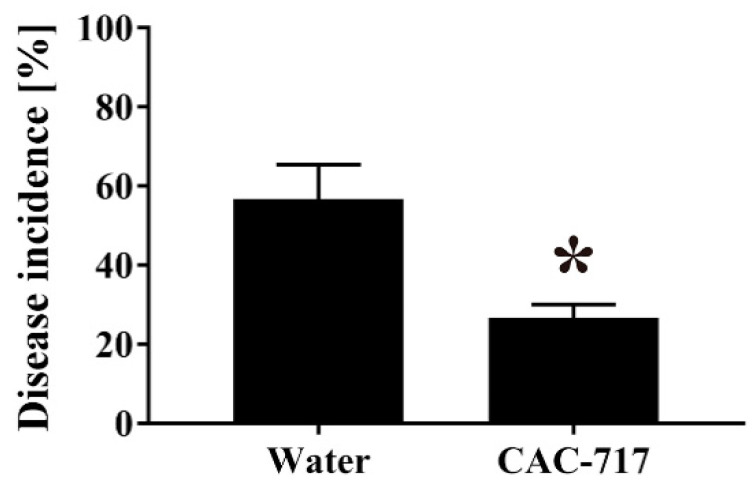
Reduced disease incidence in *Xcc*-contaminated seeds after CAC-717 treatment. Cabbage seeds contaminated with *Xcc* were treated with distilled water or CAC-717 at 25 °C for 30 min. The seeds were then cultured at 25 °C for 5 days, and the incidence of disease in the seeds was evaluated as described in Methods. Data shown as mean ± SEM of triplicates and are representative of two independent experiments; * indicates a significant difference (*p* < 0.05) between the two groups by Mann–Whitney U-test.

Next, the contaminated seeds were subjected to treatment with CAC-717 or distilled water at 25 °C for 30 min. In terms of viable cell number, 30 min treatment with CAC-717 at 25 °C had a clear disinfection effect on cabbage seeds contaminated with *Xcc* as compared with those treated with distilled water (Figure 3). The bacterial cell number was significantly lower after CAC-717 treatment (0.36 log_10_ CFU/mL (SEM = 0.23 log_10_ CFU/mL)) than after distilled water treatment (3.52 log_10_ CFU/mL (SEM = 0.12 log_10_ CFU/mL)). Following 5 days of cultivation at 25 °C, the incidence of disease was 56.67 ± 8.82% after distilled water treatment compared with 26.67 ± 3.33% after 30 min of CAC-717 treatment, and the difference was significant (*p* < 0.05) (Figure 4).

**Figure 5:**
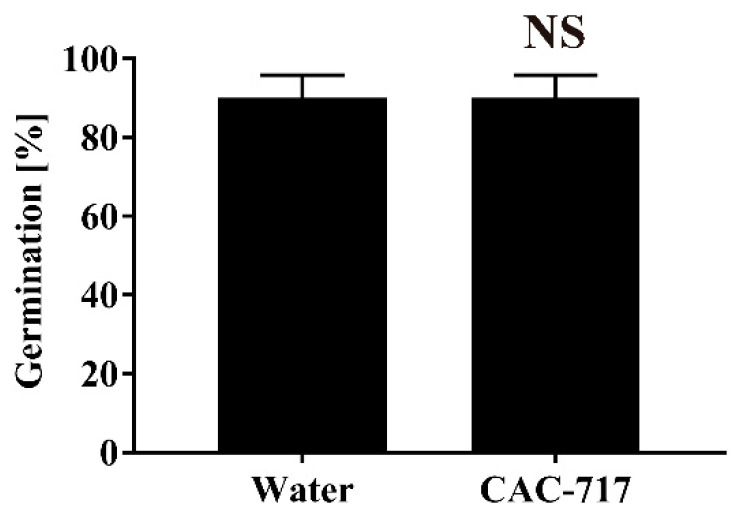
No change in germination rate of non-contaminated cabbage seeds after CAC-717 treatment. Non-contaminated cabbage seeds were treated with distilled water or CAC-717 at 25 °C for 30 min. The germination rate was then measured as described in Methods. Data shown as mean ± SEM of triplicates and are representative of two independent experiments. There was no significant difference (NS) between the two groups by Mann–Whitney U-test.

**Figure 6:**
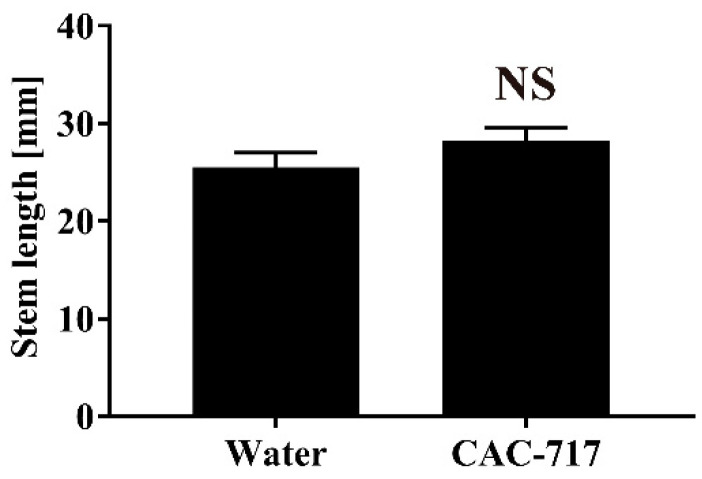
No change in plant stem length of non-contaminated cabbage seeds after CAC-717 treatment. Non-contaminated cabbage seeds were treated with distilled water or CAC-717 at 25 °C for 30 min. Plant stem length was then measured as described in Methods. Data shown as mean ± SEM of triplicates and are representative of two independent experiments. There was no significant difference (NS) between the two groups by Mann–Whitney U-test.

Finally, CAC-717 was used to treat non-contaminated seeds, and the germination rate and plant stem length were measured. We found that the germination rate was 90.00 ± 5.77% after 30 min of distilled water treatment at 25 °C and 90.00 ± 5.77% after 30 min of CAC-717 treatment at 25 °C, indicating no significant difference between CAC-717 and control treatment (*p* > 0.05) (Figure 5). In addition, the plant stem length of germinated and grown cabbage, as measured by image data analysis software (ImageJ v. 1.52a), was 25.52 ± 1.49 mm after 30 min of distilled water treatment at 25 °C and 28.27 ± 1.27 mm after 30 min of CAC-717 treatment at 25 °C, again indicating that there was no significant difference between CAC-717 and distilled water treatment (*p* > 0.05) (Figure 6).

## 4. Discussion

Black rot is one of the most common and devastating diseases of brassica crops worldwide [2], resulting in the extensive loss of cabbage and other cruciferous plants. The disease is mainly caused by *Xcc* infection [1], and although there are several methods for seed disinfection of seed-borne pathogens such as *Xcc*, including chemicals such as calcium hypochlorite [7,8] and eugenol [18], as well as pulsed electric field [19], plasma [20,21], and ozone [21], they all have various drawbacks, such as low efficacy, environmental impacts, public safety risks, or reduced germination.

Here, we approached the disinfection of *Xcc*-contaminated seeds using CAC-717, a new type of disinfectant containing calcium hydrogen mesoscopic crystals. Our results show that 30 min of CAC-717 treatment at 25 °C is sufficient to inactivate *Xcc* and disinfect *Xcc*-contaminated seeds, as well as reduce the incidence of black rot.

Regarding the bactericidal effect of CAC-717 on *Xcc*, in vitro analysis demonstrated a *D*-value of 0.319 min, which was much shorter than 2.137 min for the *D*-value of hot water (50 °C). Thus, effective disinfection was achieved in a very short time compared to the conventional method. CAC-717 treatment of *Xcc*-contaminated seeds also led to a significant reduction in viable bacterial cell number, and a significant decrease in the incidence of black rot. These findings confirm the effectiveness of CAC-717 treatment on *Xcc*-contaminated seeds. Furthermore, CAC-717 treatment of non-contaminated seeds had no significant effect on either the germination rate or plant stem length. Therefore, seed disinfection by CAC-717 does not seem to adversely affect seed germination or growth.

Taken together, our results suggest that CAC-717 is a useful disinfectant for *Xcc*-contaminated seeds. However, there are a number of limitations in this study. First, because a single isolate was used in the present study, the effectiveness of CAC-717 on other isolates of *Xcc* as well as other seed-borne bacteria remains unclear. However, it should be noted that our previous reports have shown that bacteria such as *Salmonella* and *E. coli* can also be inactivated by CAC-717 [10], suggesting that CAC-717 may be effective against a broad range of bacteria including other isolates of *Xcc*. In addition, it remains unclear whether CAC-717 is effective as a treatment for other varieties of *Brassica oleracea* beside cabbage. Therefore, further studies on CAC-717 using various isolates of *Xcc* and a variety of seeds including *Brassica oleracea* are required. Additionally, CAC-717 treatment was conducted at 25 °C throughout this study, but the effectiveness of the treatment may be temperature dependent. Furthermore, as only a small quantity of seeds was used in the present study, it remains unclear whether CAC-717 will have a similar efficiency for larger batches. Therefore, further studies on the relationship between seed batch size and inactivation efficiency as well as the optimization of treatment time and temperature are required. In addition, there are a variety of conventional disinfection methods for seeds, including hot water (50 °C) treatment. Comparison of the effectiveness of these methods with CAC-717 treatment, as well as the potential use of combination treatments, needs to be investigated in terms of disinfection efficiency as well as germination rate using seed-borne bacteria-contaminated seeds. This is important because the germination test is unrelated to the emergence test. These tests will be conducted in a future study. Further studies on this issue will lead to the development of more effective disinfection procedures. Finally, the mechanisms by which CAC-717 inactivates *Xcc* remains unclear. Our previous studies showed that CAC-717 destroys the genomic DNA of bacteria such as *E. coli* [10] and *S. enterica* [10]. Membrane disruption by CAC-717 can expose genomic DNA to digestion by DNase. However, it remains unclear whether the DNA damage contributes to the mechanism of disinfection by CAC-717 or whether this is merely a consequence of bacterial cell death. Interestingly, recent experiments show that a prion agent, which is a pathogen that lacks DNA, can be inactivated by CAC-717 [13]. These findings suggest that the presence of DNA is not essential for the inactivation mechanism of CAC-717. Hence, further studies on the mechanisms by which CAC-717 inactivates *Xcc* are required.

## 5. Conclusions

CAC-717 treatment can be expected to be a technology that is capable of disinfection without adversely affecting seed germination or growth. CAC-717 is likely to be useful as an efficient disinfectant for seeds. However, further optimization of the treatment conditions, such as treatment temperature and treatment time, for seed disinfection by CAC-717 as well as larger seed batches and seed variety may be necessary for practical application of this technology.
